# Association between Parkinson’s disease and cardiovascular disease mortality: a prospective population-based study from NHANES

**DOI:** 10.1186/s12944-024-02200-2

**Published:** 2024-07-04

**Authors:** Li Ke, Lei Zhao, Wenli Xing, Qiaosheng Tang

**Affiliations:** 1 Department of Neurology, Suining Central Hospital, Suining, Sichuan Province China; 2 Department of Neurology, Nanxishan Hospital, Nanning, Guangxi Zhuang Autonomous Region China

**Keywords:** NHANES, Cardiovascular disease mortality, Parkinson’s disease

## Abstract

**Background and aim:**

Conflicting results have been reported on the association between Parkinson's disease (PD) and cardiovascular disease (CVD) mortality in different populations. Therefore, studying the relationship between PD and CVD mortality is crucial to reduce mortality caused by the former.

**Methods:**

In this cohort investigation, we enrolled 28,242 participants from the National Health and Nutrition Examination Survey spanning from 2003 to 2018. The 380 cases of PD in the cohort were identified by documenting ‘ANTIPARKINSON AGENTS’ in their reported prescription medications. Mortality outcomes were ascertained by cross-referencing the cohort database with the National Death Index, which was last updated on 31 December 2019. Cardiovascular disease mortality was categorised according to the 10th revision of the International Classification of Diseases by using a spectrum of diagnostic codes. Weighted multivariable Cox regression analysis was used to examine the association between PD and the risk of CVD mortality.

**Results:**

A total of 28,242 adults were included in the study [mean age, 60.156 (12.55) years, 13,766 men (48.74%)], and the median follow-up period was 89 months. Individuals with PD had an adjusted HR of 1.82 (95% CI, 1.24–2.69; *p* = 0.002) for CVD mortality and 1.84 (95% CI, 1.44–2.33; *p* < 0.001) for all-cause mortality compared with those without PD. The association between PD and CVD mortality was robust in sensitivity analyses, after excluding participants who died within 2 years of follow-up and those with a history of cancer at baseline [HR,1.82 (95% CI, 1.20–2.75; *p* = 0.005)].

**Conclusions:**

PD was associated with a high long-term CVD mortality rate in the US population.

## Introduction

Parkinson’s disease (PD) is a sophisticated and progressively worsening neurodegenerative condition and characterised by degeneration of dopaminergic neurons in the substantia nigra and accumulation of alpha synuclein in neurons; PD includes motor symptoms, such as tremor, muscle stiffness, reduced mobility and balance, and non-motor features such as cognitive decline, depression and pain [1]. Although PD is relatively rare in individuals under the age of 50 years, the prevalence and burden of PD increase rapidly worldwide as life expectancy increases. The 2019 Global Burden of Disease, Injury and Risk Factors (GBD) study estimated that more than 8.5 million people worldwide suffer from PD [2]. The global burden of PD is expected to exceed 17 million cases by 2040 [3]. Therefore, evaluating serious complications that are caused and actively intervened by PD is crucial to reduce the disability and mortality rates of PD.

Although epidemiologic studies have consistently reported that PD is associated with higher premature mortality rates than the general population [4], the association between PD and cardiovascular diseases (CVDs) remains uncertain. Previous studies suggested that the incidence of CVD is reduced in people with PD [5, 6], and a lack of dopamine may prevent ischaemic damage to the brain by reducing the effects of excitatory toxicity. However, patients with PD have a higher risk of MI, ischaemic stroke, CHF and all-cause mortality than those without PD [7]. In the United States, CVDs including myocardial infarction (MI), ischaemic stroke and congestive heart failure (CHF) account for more than 25% of deaths [8]. Therefore, exploring the relationship between PD and CVD mortality among adult Americans is important.

In this study, we utilised a substantial cohort with extensive, long-term follow-up data from the National Health and Nutrition Examination Survey (NHANES) to evaluate CVD mortality rates and overall mortality rates among individuals with PD.

## Methods

### Study population

**Fig. 1:**
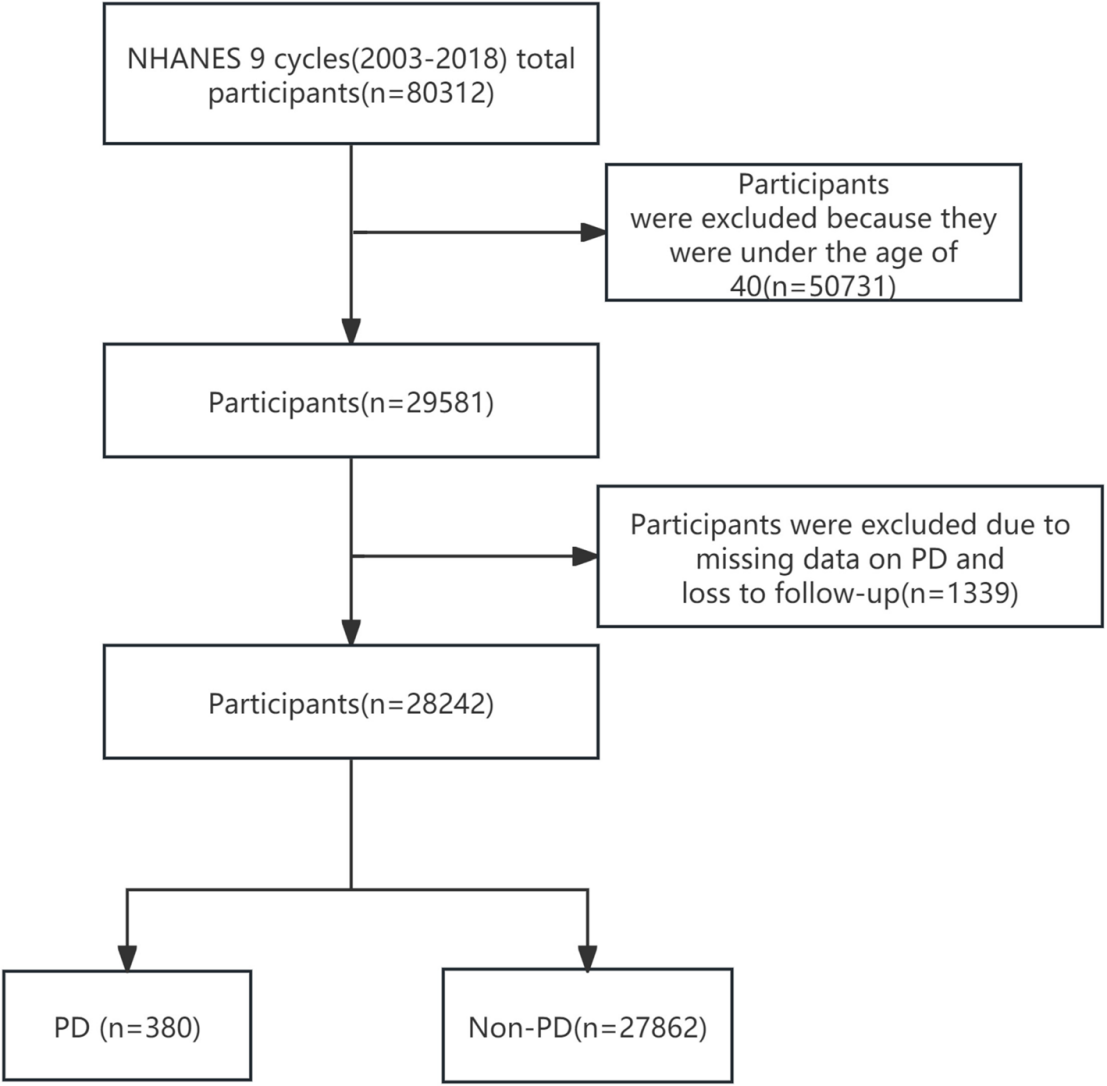
Study flow chart

From 2003 to 2018, 80,312 participants were involved in the NHANES study. Participants under 40 years of age were excluded based on the epidemiological characteristics of PD. After removing participants with missing data on PD and loss to follow-up, statistical analysis was performed on 28,242 participants, which included 380 participants with PD and 27,862 participants without PD. The complete process of data integration is illustrated in Fig. 1.

The National Centre for Health Statistics (NCHS) conducts NHANES, a national representative study, to evaluate the health or nutritional state of the US population that is not institutionalised. Utilising a multistage, stratified probability sampling strategy, NHANES collects demographic and detailed health information through home visits, screening, and laboratory testing, by a mobile exam centre. The NCHS Research Ethics Review Board approved the NHANES study protocol, and participants provided written informed permission at enrolment (source: https://www.cdc.gov/nchs/nhanes/irba98.htm). The Suining Central Hospital institutional review board deemed the study exempt due to its utilisation of publicly accessible and deidentified data, thus waiving the requirement for informed consent. This study adhered to the Strengthening the Reporting of Observational Studies in Epidemiology (STROBE) guidelines to maintain high-quality reporting.

### Assessment of PD and mortality

In the NHANES database, participants with PD were identified by the presence of ‘ANTIPARKINSON AGENTS’ in their prescription medication responses [9–11]. Individuals had to be receiving treatment for PD to be classified as having the disease due to limitations in NHANES medications and codes. Others were categorised as non-Parkinson’s disease participants. Mortality data were collected by linking the cohort database to the Centres for Disease Control’s National Death Index as of 31 December 2019. Cardiovascular mortality in this analysis encompasses a range of ICD codes, specifically: I00–I09 for acute rheumatic fever and chronic rheumatic heart conditions; I11 for hypertensive heart disease; I13 for combined hypertensive heart and renal disease; I20–I25 for ischemic heart diseases; I26–I28 for pulmonary embolism and other acute pulmonary heart conditions; I29 for a variety of CVDs due to diverse causes; I30–I51 for additional forms of heart disease; and I60–I69 for cerebrovascular disorders.

### Assessment of covariables

Potential covariables included age, gender, marital status, race/ethnicity, education level, family income, body mass index (BMI), smoking status, alcohol drinking status, diabetes and hypertension [7, 12]. Race/ethnicity was classified as non-Hispanic white, non-Hispanic black, Mexican American or others. Marital status was defined as married, living with a partner or living alone. Educational attainment was divided into three categories: fewer than nine years, nine to 12 years and more than 12 years. According to a US government report, family income was divided into three categories based on the poverty income ratio (PIR): low (PIR ≤ 1.3), medium (PIR > 1.3 to 3.5) and high (PIR > 3.5). Smoking status was classified as ‘never’ (smoked fewer than 100 cigarettes), ‘former’ (smoked more than 100 cigarettes in life and smoke not at all now) and ‘now’ (smoked moth than 100 cigarettes in life and smoke some days or every day). The classification of alcohol consumption included the categories of ‘never’ (having never consumed alcohol in their lifetime), ‘former’ (having previously consumed alcohol but no longer do), ‘heavy’ alcohol use (≥ 3 drinks daily for women, ≥ 4 drinks daily for men, or binge drinking [≥ 4 drinks in one occasion for women, ≥ 5 drinks in one occasion for men] on 5 or more days in a month), ‘moderate’ alcohol use (≥ 2 drinks daily for women, ≥ 3 drinks daily for men, or binge drinking on ≥ 2 days in a month, or a history of daily binge drinking) and ‘mild’ alcohol use (not meeting the criteria mentioned above) [13]. Physical activity (PA) was defined as the time individuals spent in engaging in activities such as walking, biking, household chores, work-related tasks, and recreational pursuits throughout the week; if exercise was not conducted in a week, then the exercise time was 0. Previous diseases, including hypertension, diabetes, stroke, and coronary heart disease, were identified through participants’ responses to the questionnaire regarding whether a doctor had been notified of these conditions in the past.

### Statistical analysis

This study conducted a secondary analysis of publicly available data from the NHANES dataset. Sampling weights and design variables were used in all analyses to avoid biased estimates and inflated significance levels. Therefore, our analysis followed the NHANES guidelines by incorporating a complex sampling design and sampling weights [14]. Our research data were derived from family interviews and Mobile Examination Centre (MEC) data collected during NHANES surveys. As per the NHANES survey sample weight analysis guidelines, weights provided by MEC should be utilised. Sampling weight was calculated by taking the MEC weight for each participant and multiplying it by 1/8 × 2 years, spanning from 2003 to 2018. The National Death Index is updated every 4 years, and the latest follow-up data are currently available as of 31 December 2019. The follow-up period for each participant was calculated from the date of testing at the MEC to the date of death or the end of follow-up on 31 December 2019. Considering the small percentage of missing data for all variables (missing rates ranged from 0 to 9%), we employed a multivariable single imputation method using an iterative imputer with a Bayesian Ridge model as the estimator at each imputation step, following the approach proposed by van Buuren and Groothuis–Oudshoorn (2011). Categorical and continuous variables were presented as unweighted percentages and means (standard deviation [SD]). The study utilised linear regression analyses and Chi-square tests to compare continuous and categorical variables, respectively. Weighted multivariable Cox proportional hazards regression models were employed to assess the hazard ratio (HR) and 95% confidence interval (95% CI) for the relationship between PD and the risks of CVD and all-cause mortality. Model 1 was adjusted for age, sex, marital status, race/ethnicity, education level, family income and NHANES cycle. Model 2 included additional adjustments for smoking status, alcohol drinking status and physical activity. Finally, Model 3 was further adjusted for BMI, diabetes and hypertension [11, 12]. Sensitivity analyses were performed to assess the reliability of our findings. To mitigate the risk of reverse causality, we excluded individuals who passed away within 2 years of recruitment. Participants with cancer were also excluded to prevent any potential impact on mortality rate [15].

To analyse the association between PD and CVD mortality based on general characteristics, subgroup analysis was conducted to analyse gender (male vs. female), age (< 50 years vs. ≥ 50 years), race/ethnicity (non-Hispanic white vs. other), BMI (< 30 kg/m^2^ vs. ≥ 30 kg/m^2^), smoking status (never compared with before or now) and drinking status (never compared with before or now) by using a multivariable Cox proportional hazards regression model. PD is considered an age-related condition, with the incidence and prevalence rates increasing steadily with age. When PD occurs in individuals under the age of 50 years, it is referred to as early-onset PD. Subgroup analysis often uses 50 years old as the age threshold [16]. Research indicated a link between being underweight and a higher likelihood of developing PD [17]. Additionally, a clear negative correlation exists between BMI levels at the time of diagnosis and mortality rates among individuals with PD [18]. Obesity, defined as having a BMI ≥ 30 kg/m^2^, is associated with high mortality rates from heart disease and stroke [19]. As a result, a BMI of 30 kg/m^2^ was utilised as the cut-off point for subgroup analysis. The covariates were adjusted in the same way as in Model 3. Likelihood ratio testing was used to investigate the interactions of the subgroups. All analyses were performed using statistical software packages R4.3.3 (http://www.R-project.org) and Free Statistics software version 1.9.2 (Beijing Free Clinical Medical Technology Co., Ltd.).

## Results

### Baseline characteristics

At baseline, 380 participants had PD, whereas 27,862 did not. Table 1 shows the baseline characteristics of the 28,242 study participants. The mean (SD) age of the participants was 60.1 (12.5) years, and 13,766 (48.7%) were men and 14,476 (51.2%) were women. In comparison with the 27,862 individuals without PD, the 380 individuals with PD were more likely to be older ( 60.092(12.539) years vs 64.829 (12.955) years respectively), have a higher BMI (30.566 (7.422) kg/m^2^ vs. 29.382 (6.666), respectively) and they were more likely to have a higher prevalence rate of diabetes (17,090.00 (25.47%) vs.128.00 (33.68%), respectively) and hypertension [15736.00 (56.49%) vs. 259.00 (68.16%), respectively].

**Table 1:** Baseline characteristics of participants in the NHANES 2003–2018 cycles

Characteristic	Participants^a^				
	Overall	Non-PD	PD	*p*	
	*n* = 28,242	*n* = 27,862	*n* = 380		
Age	60.15 (12.55)	60.09 (12.53)	64.82 (12.95)	< 0.0001	
Sex					
Male	13,766.00 (48.74)	13,589.00 (48.77)	177.00 (46.58)	0.3955	
Female	14,476.00 (51.26)	14,273.00 (51.23)	203.00 (53.42)		
Race					
Non-Hispanic White	12,763.00 (45.19)	12,515.00 (44.92)	248.00 (65.26)	< 0.0001	
Non-Hispanic Black	6107.00 (21.62)	6052.00 (21.72)	55.00 (14.47)		
Mexican American	4163.00 (14.74)	4126.00 (14.81)	37.00 ( 9.74)		
Oher	5209.00 (18.44)	5169.00 (18.55)	40.00 (10.53)		
Marital					
Married or living with partners	17,361 (61.51)	17,164(61.64)	197.00 (51.84)	0.0001	
Living alone	10,863.00 (38.49)	10,680 .00(38.36)	183.00(48.16)		
PIR					
≤ 1.30	7566.00 (29.50)	7432.00 (29.37)	134.00 (38.95)	< 0.0001	
1.31–3.50	9806.00 (38.24)	9670.00 (38.22)	136.00 (39.53)		
> 3.50	8273.00 (32.26)	8199.00 (32.41)	74.00 (21.51)		
Education					
Less than high school	8008.00 (28.40)	7883.00 (28.34)	125.00 (32.89)	0.1305	
High school or equivalent	6555.00 (23.25)	6476.00 (23.28)	79.00 (20.79)		
Above high school	13,635.00 (48.35)	13,459.00 (48.38)	176.00 (46.32)		
Smoke					
Never	14,457.00 (51.23)	14,280.00 (51.29)	177.00 (46.70)	0.0809	
Former	8518.00 (30.18)	8402.00 (30.18)	116.00 (30.61)		
Now	5247.00 (18.59)	5161.00 (18.54)	86.00 (22.69)		
Alcohol					
Never	3850.00 (15.47)	3802.00 (15.48)	48.00 (15.00)	< 0.0001	
Former	5422.00 (21.79)	5319.00 (21.66)	103.00 (32.19)		
Mild	8929.00 (35.89)	8814.00 (35.89)	115.00 (35.94)		
Moderate	3257.00 (13.09)	3232.00 (13.16)	25.00 ( 7.81)		
Heavy	3422.00 (13.75)	3393.00 (13.82)	29.00 ( 9.06)		
Physical activity, min/week	583.95(1202.83)	587.54 (1206.58)	321.06(846.77)	< 0.0001	
BMI/kg.m2	29.39 (6.67)	29.38(6.66)	30.56 (7.42)	0.0027	
Hypertension					
No	12,241.00 (43.35)	12,120.00 (43.51)	121.00 (31.84)	< 0.0001	
Yes	15,995.00 (56.65)	15,736.00 (56.49)	259.00 (68.16)		
Diabetes					
No	21,001.00 (74.42)	20,749.00 (74.53)	252.00 (66.32)	0.0003	
Yes	7218.00 (25.58)	7090.00 (25.47)	128.00 (33.68)		

*Abbreviation*: *NHANES* National Health and Nutrition Examination Survey

^a^Data are presented as unweighted number for categorical variables (percentage) and continuous variables (mean ± standard error)

### PD and mortality

**Fig. 2:**
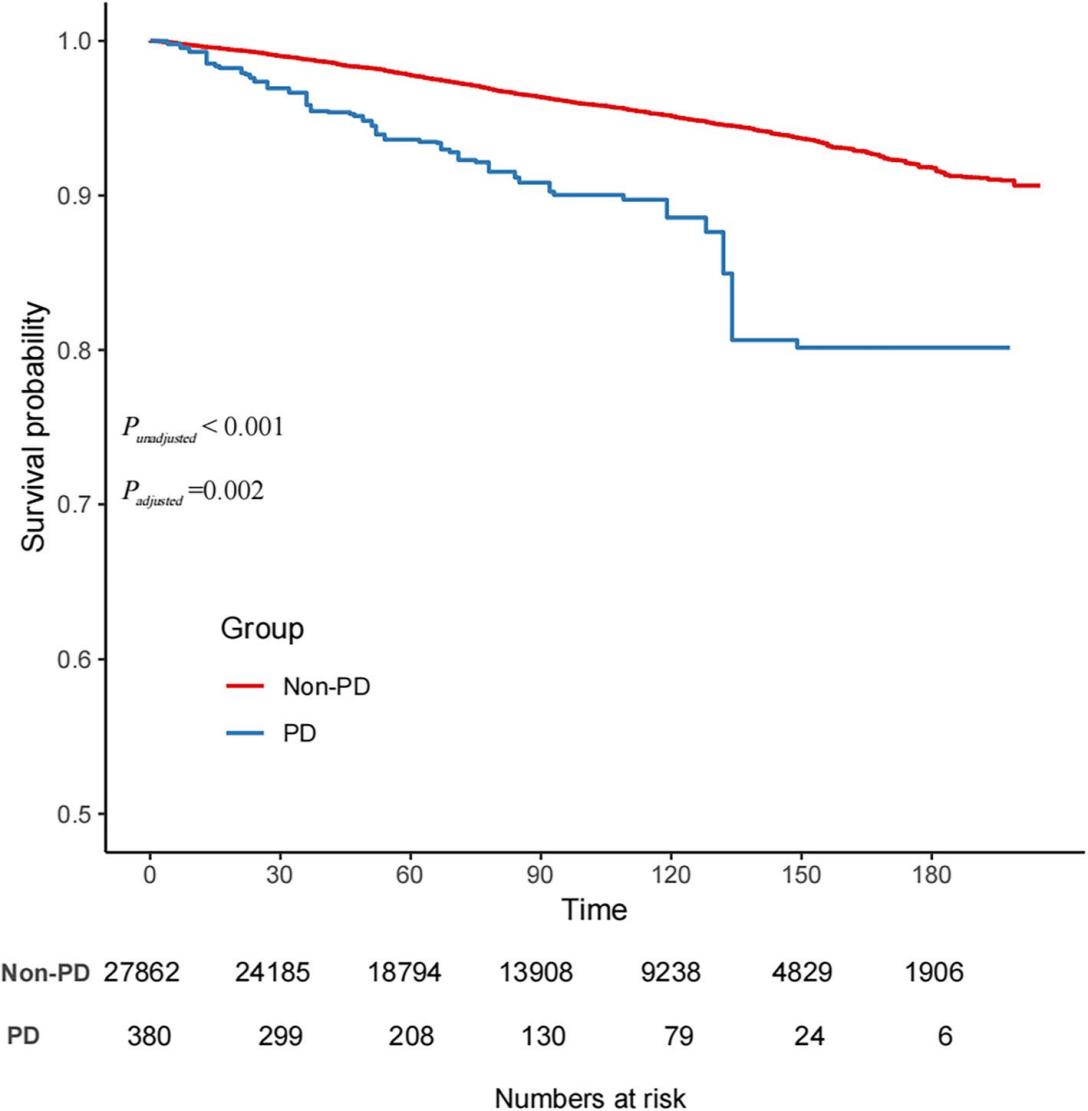
Kaplan–Meier survival curve for CVD mortality in people with and without PD

During 1–16 years of follow-up of NHANES 2003–2018, a total of 5,268 all-cause deaths and 1.658 CVD deaths were identified. The median follow-up period was 89 months. Individuals with PD had an adjusted HR of 1.82 (95% CI, 1.24–2.69; *p* = 0.002) for CVD mortality and 1.84 (95% CI, 1.44–2.33; *p* < 0.001) for all-cause mortality compared with those without PD (Table 2). Figure 2 displays the Kaplan–Meier curve for CVD mortality and the observed survival rates in the PD group compared with those in the non-PD group. Hence, the observed survival in the PD group was significantly lower than that in the non-PD population.

**Table 2:** Hazard ratios of CVD and all-cause mortality by PD among adults in NHANES 2003–2018

	Deaths, no./total no			
	Without PD	With PD	HR (95% CI)	*P_value*
All-cause mortality				
Crude model	5133/27862	135/380	2.44(1.84, 3.25)	< 0.001
Mode 1	5133/27862	135/380	1.84(1.43, 2.37)	< 0.001
Model 2	5133/27862	135/380	1.85(1.47, 2.33)	< 0.001
Model 3	5133/27862	135/380	1.84(1.44, 2.33)	< 0.001
CVD mortality				
Crude model	1616/27862	42/380	2.44(1.97, 4.11)	< 0.001
Mode 1	1616/27862	42/380	1.95(1.32, 2.89)	< 0.001
Model 2	1616/27862	42/380	1.87(1.27, 2.73)	< 0.001
Model 3	1616/27862	42/380	1.82(1.24, 2.69)	0.002

Model1: Adjusted for age, sex, marital status, race/ethnicity, education level, family income, and NHANES cycle

Model2: Further adjusted for smoking status, Physical activity and alcohol drinking status

Model3: Further adjusted for BMI, hypertension, diabetes

### Subgroup analyses

**Fig. 3:**
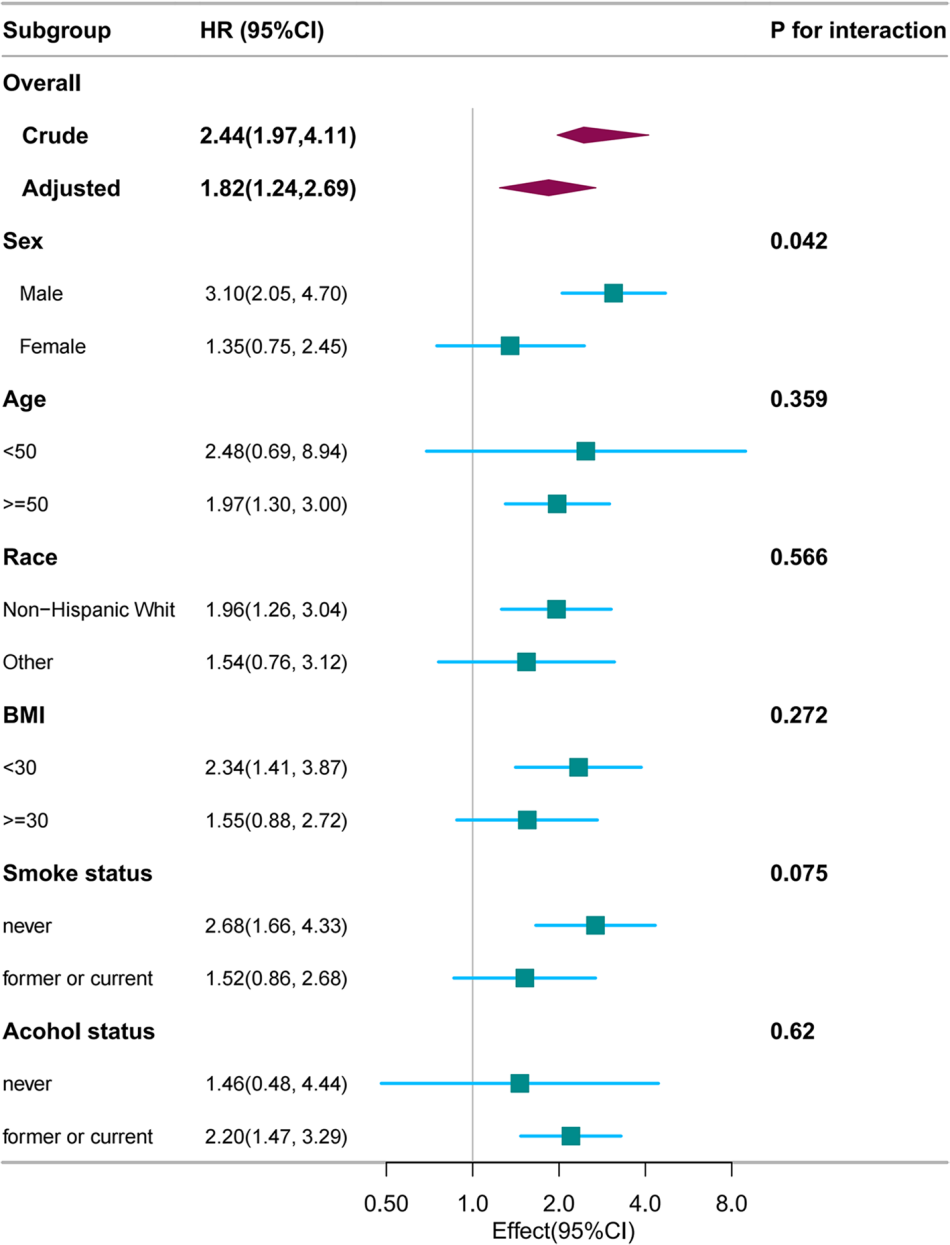
Association between Parkinson's disease and CVD mortality according to general characteristics. The stratifications were adjusted for all variables (education level, marital status, family income, NHANES cycle, physical activity, hypertension and diabetes except for the stratification factor itself. Squares represent the HRs and horizontal lines represent 95% CIs. Diamonds represent the overall HR, and the outer points of the diamonds represent the 95% CI. BMI, body mass index; CI, confidence interval; CVD, cardiovascular disease; HR, hazard ratio

Figure 3 presents the results of subgroup analyses. PD was associated with CVD mortality in participants who were aged ≥ 50 years (HR, 1.96; 95% CI, 1.26–3.04), non-Hispanic White (HR, 1.96; 95% CI, 1.26–3.04), male (HR,3.10; 95% CI, 2.05–4.70), those with a BMI < 30 kg/m2 (HR, 2.34; 95% CI, 1.41–3.87), never smoking (HR, 2.68; 95% CI, 1.66–4,33) and former or current alcohol drinkers (HR, 2.20; 95% CI, 1.47–3.29). PD was not associated with PD and CVD mortality in females, aged < 50 years, other races/ethnicities, those with a BMI ≥ 30 kg/m2, former or current smokers and never drinkers.

### Sensitivity analysis

The results of sensitivity analyses are presented in Table 3. After excluding participants who died within 2 years of follow-up and those with a history of cancer at baseline, the adjusted Hazard Ratio (HR) for CVD mortality was 1.82 (95% CI, 1.20–2.75; *p* = 0.005) and the all-cause mortality was 1.77 (95% CI, 1.38–2.26; *p* < 0.001) for individuals with Parkinson's Disease (PD) compared with those without PD.

**Table 3:** Sensitivity analyses, excluding those with cancer and died within 2 years of follow-up

	Deaths, no./total no			
	Without PD	With PD	HR (95% CI)	*P_value*
All-cause mortality				
Crude model	3273/22705	82/273	2.44(1.84, 3.25)	< 0.001
Mode 1	3273/22705	82/273	1.84(1.43, 2.37)	< 0.001
Model 2	3273/22705	82/273	1.73(1.36, 2.20)	< 0.001
Model 3	3273/22705	82/273	1.77(1.38, 2.26)	< 0.001
CVD mortality				
Crude model	1069/22705	30/273	2.71(1.80, 4.08)	< 0.001
Mode 1	1069/22705	30/273	1.91(1.23, 2.95)	0.004
Model 2	1069/22705	30/273	1.77(1.17, 2.68)	0.007
Model 3	1069/22705	30/273	1.82(1.20, 2.75)	0.005

Model1: Adjusted for age, sex, marital status, race/ethnicity, education level, family income, and NHANES cycle

Model2: Further adjusted for smoking status, Physical activity and alcohol drinking status

Model3: Further adjusted for BMI, hypertension, diabetes

## Discussion

This cohort study's findings indicate that PD elevates the risk of CVD mortality and overall mortality. The robustness of these results was confirmed through subgroup and sensitivity analyses.

Our findings on overall mortality rate are consistent with previous research findings. A historical cohort study spanning 11 years revealed a mortality rate of 1.64 (95% CI: 1.21–2.23) among patients with PD compared with the control group [20]. Similarly, the Sydney and Netherlands multicentre study reported a higher mortality rate in individuals with PK compared to population data [21, 22]. A meta-analysis concluded that cognitive impairment/dementia, ageing, late age of onset, male and gait disturbance are risk factors for mortality in patients with PD [23].

Research on CVD mortality in people with PD remains limited and controversial. A previous study showed that the risk of ischemic heart disease in patients with PD remains unchanged compared with that in the general population (HR 1.1, 95% CI 0.62.0) [24]. Even some studies suggested that people with PD have a reduced overall incidence of ischaemic stroke and heart attack [25, 26]. Individuals with PD may encounter autonomic dysfunction, cardiomyopathy, coronary heart disease, arrhythmia or sudden cardiac death (SCD), resulting in a high prevalence of heart failure [27, 28]. In a recent study by Park et al. [11]. in South Korea, a nationwide cohort analysis revealed that individuals with PD may face a greater probability of experiencing cardiovascular events and death compared with those without the condition; individuals with PD had a higher risk of myocardial infarction (HR 1.43,95% CI:1.28–1.59), ischemic stroke (HR 1.42,95% CI:1.31–1.54]) and congestive heart failure (HR 1.65,95% CI:1.52–1.78). Our research findings also indicate that the CVD mortality rate among patients with PD is higher than those in non-Parkinson’s patients. By utilising a substantial sample size of American participants, our study contributes to enhancing the overall applicability of the results.

Autonomic dysfunction is frequently observed in PD and can manifest in the autonomic nervous system, including the heart [29]. In a study conducted on the heart tissue of Parkinson's disease patients in Japan, it was discovered that 9 out of 11 patients had Lewy bodies present in tyrosine hydroxylase positive and negative neural processes, suggesting that the postganglionic sympathetic nervous system and intrinsic neurons in the heart play a role in the development of PD [30]. A prospective study conducted in Sweden revealed that diabetes and elevated fasting blood glucose levels were identified as risk factors for PD. The study also found that a higher neutrophil to lymphocyte ratio (NLR) in the general population was linked to an increased risk of PD. Interestingly, diabetes, fasting glucose and NLR are all associated with the risk of coronary events or ischemic stroke [12]. Hence, patients with PD can develop coronary heart disease and ischemic stroke. Most patients with PD receive levodopa treatment, which has been shown to increase homocysteine levels in the blood; elevated homocysteine levels have been associated with a higher incidence of cerebrovascular and cardiovascular diseases [20]. Studies also suggest that the relationship between PD and CVD is complex, with overlapping biological mechanisms, including inflammation, insulin resistance, lipid metabolism and oxidative stress [31]. These scientific discoveries corroborate our study's perspective, suggesting that PD increases the likelihood of mortality associated with CVD.

In our stratified analysis, we identified several factors that influence the association between PD and CVD mortality, such as older age, Non-Hispanic White, male, lower BMI, never smoking and past or current alcohol consumption. Similar to a South Korea study that found a negative dose–response relationship between BMI at diagnosis and mortality in patients with PD, a 10% change in BMI was significantly linked to mortality outcomes [18]. One possible explanation for this negative correlation is that higher BMI affects insulin levels, which may play a beneficial role in dopaminergic neurodegeneration [32]. We identified a significant interaction regarding CVD mortality among individuals with PD, distinguishing between the male and female subgroups. A retrospective study analysed the trend of PD mortality revealed that males had a mortality rate twice higher than females [33]. The gender disparity in PD development could be attributed to the potential neuroprotective effect of female gonadotropins, especially circulating oestradiol, on the dopaminergic system. Men typically acquire PD at a younger age than women, leading to a higher mortality rate in men at an earlier stage, which may counterbalance other risk factors [34]. In the United States, racial and ethnic disparities exist in access to neurological care, with black and Hispanic patients being less likely than white patients to consult outpatient neurologists. This discrepancy suggests that white patients have a greater likelihood of being diagnosed with PD [35] possibly attributed to their overall higher socioeconomic status in terms of education and income compared with minority populations. Consequently, this disparity may contribute to the higher CVD mortality rates in white individuals with PD in comparison with other racial and ethnic groups. Numerous clinical studies have demonstrated a negative correlation between smoking and the occurrence of PD in both genders [36, 37]. Smoking might have a protective effect against Parkinson's disease; however, the cause of the high vulnerability to CVD mortality in patients with PD who have never smoked remains unclear.

### Study strengths and limitations

This study is the first to examine CVD mortality in patients with PD by using data from the NHANES. The sample size was both large and representative, allowing for a comprehensive analysis. However, this study has some limitations. The determination of PD within our research was reliant on participants’ self-reported medication usage, without corroboration through a formal medical diagnosis. This approach acknowledges the possibility that a subset of participants may be either unaware of their PD status or may exhibit milder symptoms not necessitating pharmacological intervention, potentially leading to an underrepresented sample. Moreover, we are mindful that patients with tremor-associated neurological conditions other than PD might be prescribed antiparkinsonian medications yet lack a definitive PD diagnosis. Such instances could precipitate misclassification within our study, thereby introducing a bias into our research outcomes. To address these limitations, future investigations should endeavour to adopt more rigorous diagnostic methodologies. This might entail comprehensive clinical evaluations by specialists in movement disorders or the employment of standardised diagnostic instruments. By integrating these refined diagnostic practices, the accuracy of PD case identification can be enhanced, thereby mitigating the risk of misclassification.

## Conclusion

This large cohort study suggests that individuals with PD have a higher risk of CVD mortality compared with those without PD. The association appears to be stronger in older age, non-Hispanic white individuals, males, those with lower BMI, non-smokers and those who currently or previously consumed alcohol. Future research should delve deeper into the biological mechanisms underlying this relationship to develop effective strategies for reducing CVD mortality in individuals with PD.

## Data Availability

No datasets were generated or analysed during the current study.
